# A comparative study of the fecal microbiota of gray seal pups and yearlings ‐ a marine mammal sentinel species

**DOI:** 10.1002/mbo3.1281

**Published:** 2022-05-23

**Authors:** Craig A. Watkins, Taylor Gaines, Fiona Strathdee, Johanna L. Baily, Eleanor Watson, Ailsa J. Hall, Andrew Free, Mark P. Dagleish

**Affiliations:** ^1^ Department of Vaccines and Diagnostics Moredun Research Institute Penicuik UK; ^2^ School of Biological Sciences University of Edinburgh Edinburgh UK; ^3^ Present address: Taylor Gaines, St. Joseph's College Craig′s Road Dumfries DG1 4UU UK; ^4^ Sea Mammal Research Unit, Scottish Oceans Institute University of St Andrews St Andrews UK; ^5^ Present address: Johanna L. Baily, Institute of Aquaculture University of Stirling Stirling FK9 4LA UK

**Keywords:** fecal microbiota, gray seal, pups, yearlings

## Abstract

Gray seals (*Halichoerus grypus*) can act as sentinel species reflecting the condition of the environment they inhabit. Our previous research identified strains of pathogenic *Campylobacter* and *Salmonella*, originating from both human and agricultural animal hosts, on rectal swabs from live gray seal (*H. grypus*) pups and yearlings on the Isle of May, Scotland, UK. We examined rectal swabs from the same pup (*n* = 90) and yearling (*n* = 19) gray seals to gain further understanding into the effects of age‐related changes (pup vs. yearling) and three different natal terrestrial habitats on seal pup fecal microbiota. DNA was extracted from a subset of rectal swabs (pups *n* = 23, yearlings *n* = 9) using an optimized procedure, and the V4 region of the 16S ribosomal RNA gene was sequenced to identify each individual's microbiota. Diversity in pup samples was lower (3.92 ± 0.19) than yearlings (4.66 ± 0.39) although not significant at the *p* = 0.05 level (*p* = 0.062) but differences in the composition of the microbiota were (*p* < 0.001). Similarly, differences between the composition of the microbiota from pups from three different terrestrial habitats (Pilgrim's Haven [PH], Rona Rocks [RR], and Tarbet Slope [TS]) were highly significant (*p* < 0.001). Pairwise tests showed significant differences between all three habitats: PH versus TS (*p* = 0.019), PH versus RR (*p* = 0.042) and TS versus RR (*p* = 0.020). This preliminary study suggests a general trend, that seal microbiomes are modified by both age and, in pups, different terrestrial habitats. Furthermore, knowledge of the microbiota species present has the potential to be used in determining the environmental quality index.

## Graphical abstract

Gray seals (*Halichoerus grypus*) can act as sentinel species reflecting the condition of the environment they inhabit. We examined rectal swabs from the pups and yearling gray seals to gain further understanding into the effects of age‐related changes and three different natal terrestrial habitats on seal pup fecal microbiota. This preliminary study suggests a general trend, that seal microbiomes are modified by both age and, in pups, different terrestrial habitats. Furthermore, knowledge of the microbiota species present has the potential to be used in determining the environmental quality index.

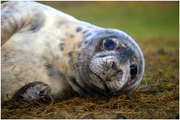

## 1 INTRODUCTION

The intestinal microbiome of terrestrial animals has been related to the health of individuals and, in sentinel species, the ecosystems they inhabit (Hanning & Diaz‐Sanchez, 2015). Marine mammals, as mesopredators, can act as sentinel species reflecting the health of coastal and marine habitats (Bik et al., 2016; Bonde et al., 2004; Cook et al., 2015; Delport et al., 2016; Gulland, 1999; Jessup et al., 2004; Moore, 2008; Nelson et al., 2013; Reddy et al., 2001; Wells et al., 2004) and this can be useful for investigating disease transmission, changes in food webs, climate change and the effects of accumulation of anthropogenic contaminants (Apprill, 2017; Baily et al., 2015; Delport et al., 2016; Godfray et al., 2019; Jepson et al., 2005). Rising levels of man‐made compounds in several marine mammal species have clearly illustrated anthropogenic pollution and its effect on marine ecosystems (Moore, 2008). Additionally, the detection in UK gray seal pup feces of specific species and strains of *Campylobacter* and *Salmonella* bacteria originating from terrestrial and anthropogenic sources implicated inadequately managed human sewage and agricultural run‐off (Baily, 2014; Baily et al., 2015, 2016).

Several studies have determined that the principal bacterial taxa inhabiting the intestines of marine mammals differ from those of terrestrial mammals and include members of the phyla Firmicutes, Bacteroidetes, Fusobacteria, Proteobacteria, and, to a lesser extent, *Actinobacteria* spp. (Banks et al., 2014; Delport et al., 2016; Glad et al., 2010; Nelson et al., 2013; Numberger et al., 2016). However, the microbiota profiles of phocid seals are further complicated by the dramatic physiological changes that seals undergo in the first year of life (Hall et al., 2001; Smith et al., 2013) which influence the intestinal microbiota composition.

Gray seals are found primarily in the northern North Atlantic Ocean, with just under 50% of their population residing in UK coastal waters (Reeves et al., 2002) and the Isle of May is the fourth largest gray seal breeding colony in the British Isles accounting for approximately 4.5% of annual pup production (Special Committee on Seals Report, 2020, http://www.smru.st-andrews.ac.uk/scos/scos-reports).

This study aimed to compare the fecal microbiota profiles of gray seal pups versus yearlings and to investigate the effects on the profiles of gray seal pups born on three different substrates within the extensively studied breeding colony on the Isle of May, Scotland, UK. The microbiota profiles were also evaluated for selected known pathogenic bacterial genera as biomarkers for use as putative indicators in an environmental quality index.

## 2 MATERIALS AND METHODS

### 2.1 Sampling

**Figure 1:**
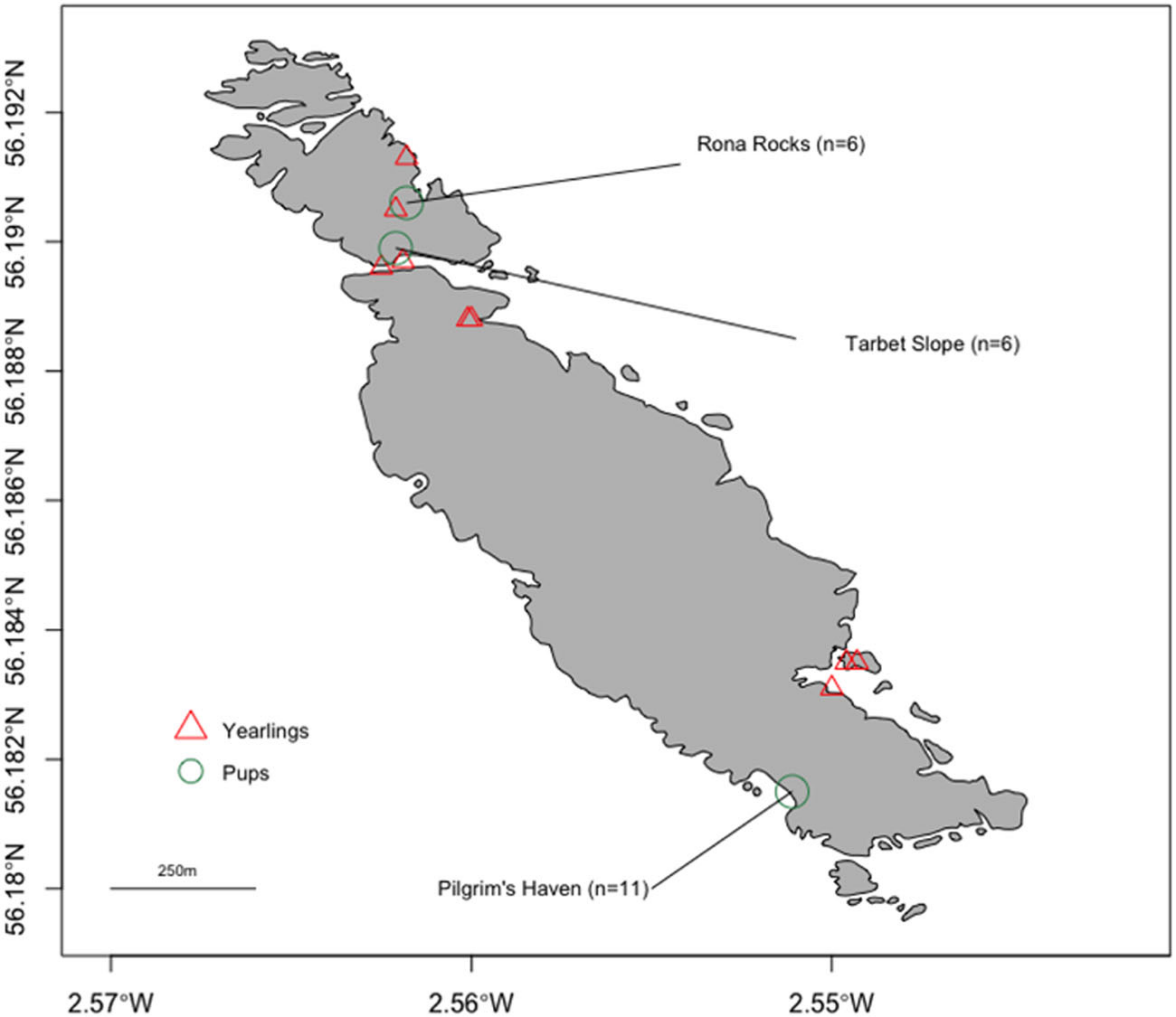
Map of seal sampling locations on the Isle of May, Scotland, UK. Circles: sampling sites of gray seal pups. Triangles: sampling sites of yearlings. Figures in parentheses are numbers of seal pups, sampled by rectal swab, from each of the three different natal terrestrial habitats that DNA was extracted successfully from. Scale bar: 250 m

Rectal swabs were taken from 90 live healthy gray seal pups and 19 live juveniles physically restrained for other on‐going studies during 6 weeks of the breeding season, in autumn 2011, on the Isle of May, a small island off the east coast of Scotland in the Firth of Forth (Figure 1). Immediately after sampling, swabs were resheathed in Amies medium with charcoal (Medical Wire & Equipment) and stored at −80°C within 12 h of sampling as described previously (Baily et al., 2015, 2016). Pups were sampled at three specific sites comprised of highly different substrates: a tidal rocky boulder beach (Pilgrim's Haven [PH], *n* = 30), rocky stagnant pools (Rona Rocks [RR], *n* = 30), and a muddy/grassy slope (Tarbet Slope [TS], *n* = 30). The yearling samples (*n* = 19) were from two areas; one in the southwest of the island separate from the pup locations and a second near Rona Rocks and south of Tarbet Slope (Figure 1).

### 2.2 Total nucleic acid extraction

Swabs, stored at −80°C, were thawed gently on ice immediately before DNA extraction using the PowerFecal DNA isolation kit (MoBio) as per the manufacturer's instructions but with modifications. Briefly, the head of each swab was cut off and placed into a DNA‐free tube containing 0.7 mm dry glass beads followed by the addition of 750 µl of bead solution, 60 µl of C1 solution, and 10 µl of proteinase K (20 mg/ml). The samples were mixed by vortexing for 2 min and incubated for 1 h at 55°C. Subsequently, samples were bead‐beaten using a MoBio vortex adapter for 10 min. and then centrifuged at 13,000*g* for 2 min. Supernatants were transferred to clean 2 ml collection tubes and 250 µl of C2 solution added and vortexed briefly, incubated at 4°C for 5 min. before centrifugation at 13,000*g* for 1 min. Resultant supernatants (600 µl) were transferred to clean collection tubes and 200 µl of C3 solution was added, vortexed briefly, and incubated at 4°C for 5 min. before centrifugation at 13,000*g* for 1 min. Then, 750 µl of supernatant was added to 1200 µl of C4 solution and vortexed for 5 s. The resultant supernatant (650 µl) was loaded into a spin filter and centrifuged at 13,000*g* for 1 min and the flow‐through discarded. After repeating this step three times (to filter a total volume of 1950 µl), the spin filters were washed with 500 µl of C5 solution and centrifuged at 13,000 *g* for 1 min. The spin filter was dried by replacing the collection tube and centrifuging for an additional 2 min at 13,000*g*. DNA was eluted by the addition of 50 µl of C6 solution to the spin filter, incubated at room temperature for 1 min and centrifuged at 13,000*g* for 1 min. DNA concentrations and quality were assessed by Nanodrop ONE™ (Thermo) and the DNA was stored at −20°C until required.

### 2.3 Polymerase chain reaction (PCR) amplification of 16S rRNA gene and next‐generation sequencing

The V4 region of the 16S rRNA gene was amplified, by PCR, from the extracted DNA as described by Caporaso et al. (2012). Briefly, a PCR master mix was prepared to contain 1× *Taq* buffer plus additional MgCl_2_ (1 mM final concentration), 0.2 mM of each of the four dNTPs, 0.25 mM of each primer (the same forward primer (515F) together with a different barcoded reverse primer (806R) were used, the reverse primer sequences differing only at the barcode region), 0.05 U/µl *Taq* DNA polymerase, and 1 ng/µl template DNA made up to a total volume of 25 µl with PCR grade water under sterile conditions. The V4 region of the 16S rRNA gene was amplified under the following conditions: 94°C for 3 min, followed by 25 cycles of 94°C for 45 s, 50°C for 60 s, and 72°C for 90 s, followed by a single cycle of 72°C for 7 min. PCR products were resolved by gel electrophoresis, gel purified, quantified using the PicoGreen assay (Promega), and stored at −20°C. Optimization of the nucleic acid extraction protocol used many of the swab samples. However, once optimized, only those DNA samples that were above a specific threshold of purity (OD 260/280 > 1.5; OD260/230 > 1.0) and produced a specific, well‐defined PCR product were selected for MiSeq sequencing: PH, *n* = 11; RR, *n* = 6; TS, *n* = 6; yearlings, *n* = 9. All the purified PCR products were pooled to make an amplicon library with each PCR product represented equally in the pool before being sent for Illumina MiSeq v2 2 × 250 bp paired‐end sequencing (Edinburgh Genomics). For swab‐only and DNA extraction kit controls, PCR products (whether visible or not) were excised from agarose gels at the expected molecular weight (400 bp), purified, and quantified before sequencing.

### 2.4 Data analysis

Illumina MiSeq sequence data were processed similarly to Watkins et al. (2021), with Quantitative Insights Into Microbial Ecology 2 (QIIME2) version 2019.4 (Bolyen et al., 2019), without read trimming based on QC plots. Demultiplexed reads were denoised and paired in QIIME2 with the DADA2 plugin (Callahan et al., 2016) and default parameters, generating a table of Amplicon Sequence Variants (ASVs). Taxonomy was assigned using a Naïve Bayes classifier trained on 99% ASV sequences extracted from the SILVA 132 database by in silico PCR with the 515F/806R primer set (Bokulich et al., 2018). Bray–Curtis dissimilarity was calculated on relative abundance tables in Primer‐E Version 6.1.12 (Primer‐E; Clark & Warwick, 2001), and ordination via nonmetric multidimensional scaling (NMDS) was used to examine beta‐diversity patterns visually. PERMANOVA and PERMDISP tests (999 permutations) on dissimilarity matrices were performed using the PERMANOVA + add‐on to Primer‐E (Anderson et al., 2008). Centered‐log ratios (CLRs) and *W* scores of differentially abundant taxa were calculated in QIIME2 on unnormalized feature tables using the analysis of compositions of microbiome (ANCOM) test plugin (Mandal et al., 2015).

## 3 RESULTS

DNA was extracted successfully from 32 (9 yearlings and 23 pups) of the 109 rectal swabs collected, as part of a previous study (Baily, 2014), and included pup rectal swabs from the three different sites on the Isle of May: PH (*n* = 11), RR (*n* = 6), and TS (*n* = 6). After filtering out low‐quality reads, 4.59M reads were analyzed with an average of 139,118 reads per sample (range 48,586–372,404), excluding controls. After denoising with DADA2 and chimera filtering, 3.29 M reads remained (38,355–254,966 per sample). Inspection of negative controls revealed no contaminant ASVs present in the swab samples; after removal of the controls, the remaining samples contained 3.21 M sequences designated into 1476 ASVs, a comparable number of ASVs to those seen in other studies using swabs (Budding et al., 2014; Stoffel et al., 2020).

Observed species rarefaction curves showed sufficient coverage of diversity in all samples (data not shown). Comparing alpha diversity between ages, the Shannon diversities (mean ± standard eror of mean [SEM] at maximum rarefaction depth of 38,350 sequences) for pup samples were lower (3.92 ± 0.19) than those for yearlings' samples (4.66 ± 0.39), although the difference did not reach statistical significance (Kruskal–Wallis test, *H* = 3.48, *p* = 0.062).

**Figure 2:**
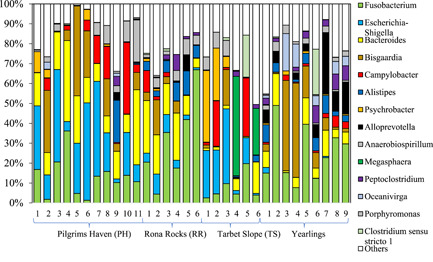
Comparison, at the genus level, of the relative composition of the fecal microbiota of pups at different geographic locations and yearlings. Taxonomic composition of microbial communities at the genus level from fecal swabs from gray seal yearling (*n* = 9) and pups (*n* = 23 in total), the latter from the three different sampling locations. Genera with <1% relative abundance across the data set were grouped as Others

Fourteen different bacterial genera were identified in pup and yearling samples at a limit of >1% abundance across the data set (Figure 2). Samples from both yearlings and pups showed high interindividual variation. Despite this, there was variation in microbiota from pup samples from the three distinct environments. The composition of many samples was dominated by the genera *Fusobacterium*, up to 67% relative abundance; *Escherichia* and/or *Shigella* (abbreviated to *Escherichia/Shigella*), up to 49%; *Bacteroides*, up to 41%, as well as significant but smaller abundances of *Bisgaardia* and *Campylobacter* identified in pups in all the three geographic locations and yearlings. Rectal swabs taken from pups at Tarbet Slope had distinct microbiota with both *Megasphaera* (Figure 2: Samples 4 and 6) and *Psychrobacter* (Figure 2: Samples 1 and 2) represented. PH Sample 9 (PH‐9), was distinctly different from the other PH samples; with little *Escherichia/Shigella* present, although *Alistipes* was well represented, similar to Sample 1 of the yearlings and RR Samples 5 and 6 (Figure 2).

The genera *Escherichia/Shigella* were not identified in the yearling group at a limit of >1% abundance except for Sample 1 (2.8%). However, *Oceaniverga* was more abundant in yearlings relative to pups (mean = 5.3% yearlings; mean = 0.2% pups). The genus *Campylobacter* was represented in all groups of pups and in yearlings (Figure 2).

**Figure 3:**
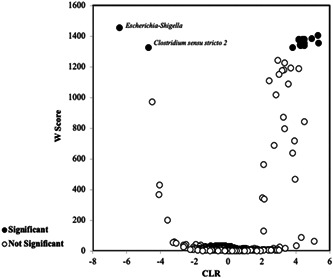
Differences in the abundance of the fecal microbiota of pups and yearlings, at the level of amplicon sequence variants. Analysis of compositions of microbiome (ANCOM) analysis of the differential abundance of amplicon sequence variants (ASVs) between gray seal pups (*n* = 23) and yearlings (*n* = 9). The centered‐log ratio (CLR) is negative for ASVs elevated in the pup samples, and positive for those elevated in the yearling samples. *W* is the ANCOM significance score, and ASVs which violate the null hypothesis, and therefore are significantly overrepresented, are shown in black‐filled circles. The ASVs which do not violate the null hypothesis (i.e., overrepresented but not significant) are shown as open circles (b)

Using ANCOM analysis, pups and yearlings were compared to identify differential biomarkers for the two age groups. The taxonomic assignments of two ASVs derived from pups, with high negative CLR difference (elevated in pups in comparison with yearlings), are shown in Figure 3 and Table 1. One of these ASVs, assigned to the genera *Escherichia/Shigella*, confirms previous analysis in Figure 2 as being significantly higher in the rectal microbiota of pups compared to yearlings. However, this ANCOM analysis also identified a low abundance ASV (<1%), assigned as *Clostridium* sensu stricto 2, as significantly elevated in pups. Thirteen ASVs were significantly elevated in yearlings, with a high positive CLR difference, as shown in Figure 3 and Table 1 also. Three Ruminococcaceae UCG‐005 and two *Fournierella* ASVs were identified as significant in yearling fecal microbiota (Table 1). The identification of the genus *Fusobacterium* (as a single sequence, Table 1), supports a similar finding in Figure 2.

**Table 1:** Differences in the abundance of the fecal microbiota of pups and yearlings, at the level of amplicon sequence variants

Taxonomy	Mean % (pups)	Mean % (yearlings)	CLR	ANCOM W score
*Escherichia–Shigella*	13.0	0.353	−6.42	1451
*Colidextribacter massiliensis*	0.015	0.946	5.31	1403
*Ruminococcaceae NK4A214* group	0.008	0.851	4.94	1383
*Fournierella*	0.0006	0.164	4.18	1378
*[Eubacterium] fissicatena* group	0.003	0.306	4.41	1377
*Ruminococcaceae UCG‐005*	0.013	0.258	4.51	1377
*Ruminococcaceae UCG‐005*	0.0009	0.237	4.17	1375
*Parasutterella*	0.003	0.440	4.28	1360
*Fecalibacterium*	0.014	0.695	4.52	1352
*Fusobacterium*	0.586	4.50	5.34	1351
*[Clostridium] innocuum* group	0.016	0.624	4.26	1339
*Oscillospira*	0.081	1.20	4.52	1338
*Ruminococcaceae UCG‐005*	0.001	0.792	4.29	1337
*Fournierella*	0.029	0.162	3.81	1325
*Clostridium* sensu stricto 2	0.958	0.033	−4.68	1324

*Note*: *Escherichia/Shigella* were not discriminated between at this level as their 16S sequences are similar. Mean % abundances of significant ASVs are calculated for gray seal pups (*n* = 23) and yearling (*n* = 9) samples.

Abbreviations: ANCOM *W*, analysis of compositions of microbiome *W* statistic; ASVs, amplicon sequence variants; CLR, centered log‐ratio.

**Figure 4:**
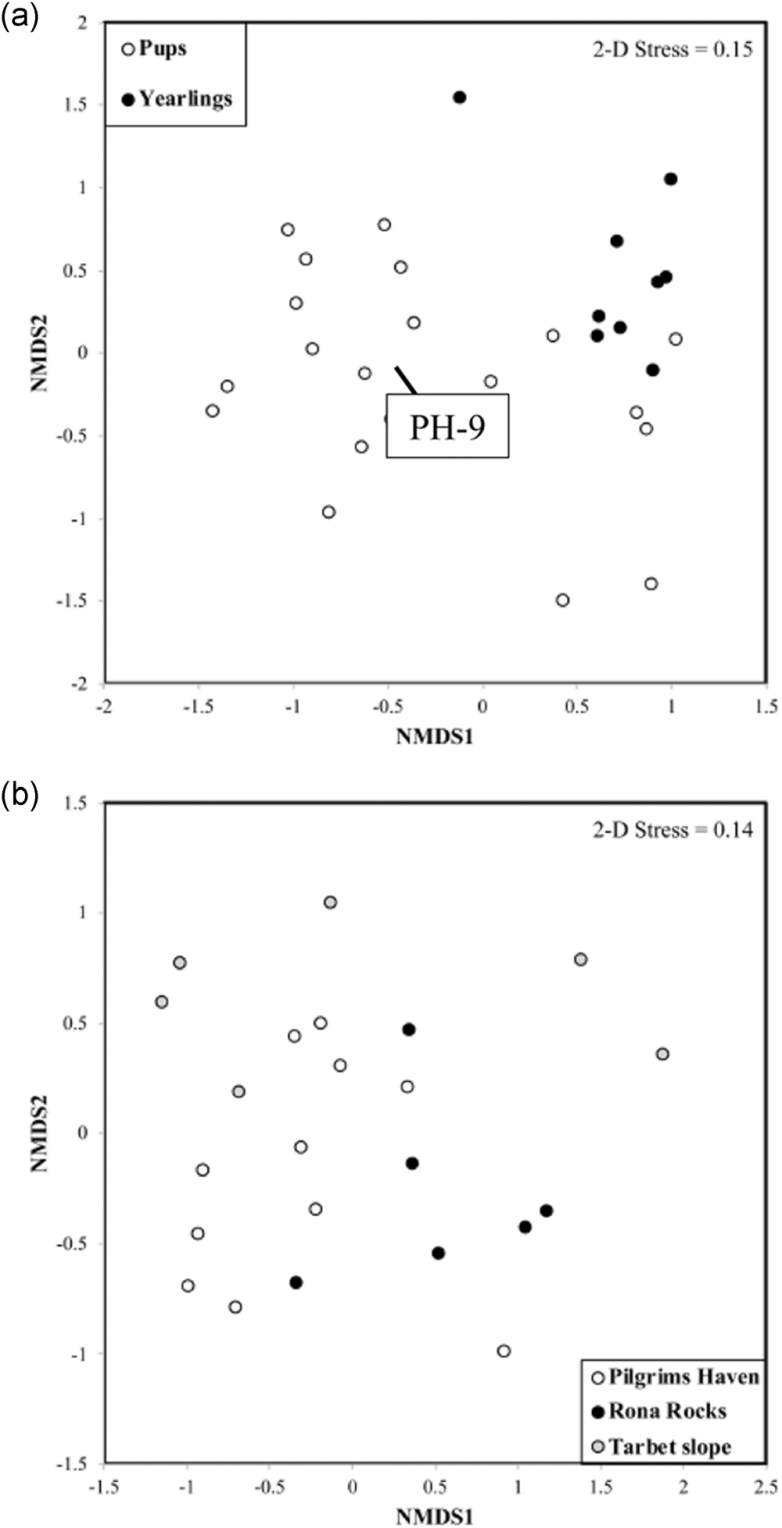
Comparisons of the similarity between the fecal microbiota of pups versus yearlings, and pups at three different natal terrestrial habitats, at the level of amplicon sequence variants. Nonmetric multidimensional scaling (NMDS) ordination plots of Bray–Curtis similarity between amplicon sequence variants (ASV) of microbial communities in seal rectal swab samples. (a) Comparison of pup and yearling samples. Note outlier, a pup sampled at Pilgrim's Haven (marked PH‐9). (b) Pup samples from the three different natal terrestrial habitats. The Kruskal stress is shown on each plot (stress < 0.2 denotes a reliable ordination)

There were significant differences in the overall composition of the microbiota present in pups versus yearlings (NMDS analysis, PERMANOVA pseudo‐*F* = 5.152; *p* < 0.001; Figure 4a) accounting for 14.7% of the total variation in the data set. The confounding factor of sex, accounting for 1.99% of the variation, was not significant (*p* = 0.901). There was significantly more variability between individual pup samples in comparison to the variability between individual yearling samples (PERMDISP *F* = 13.846; *p* = 0.004; Figure 4a). The distinct difference in the microbiota from the sample represented by PH‐9 compared with other samples within the PH group in Figure 4a confirms the result seen in Figure 2.

Differences between the composition of the microbiota in fecal swabs from pups collected from the three different locations (PH, RR, and TS) were highly significant over all natal terrestrial habitats (Pseudo‐*F* = 2.063; *p* < 0.001, 17.1% of total variation) (Figure 4b). Pairwise tests showed significant differences between all three different natal terrestrial habitats: PH versus TS (*t* = 1.446, *p* = 0.019), PH versus RR (*t* = 1.370; *p* = 0.042) and TS versus RR (*t* = 1.499; *p* = 0.020). The confounding factor of sex had no significant effect (*p* = 0.991), accounting for only 2.06% of total variation.

## 4 DISCUSSION

This study is the first investigation of the rectal microbiome in gray seals born on the Isle of May, Scotland, UK, a population studied and documented extensively over many years (Smout et al., 2011), and has shown that the composition of the microbiome differs significantly between pups and yearlings. A second finding, (although not definitive, as the soil microbiota were not analyzed at the sites where pups were sampled), suggested that the rectal microbiome of pre‐weaned pups was highly influenced by the substrate of the terrestrial habitat on which they were born, also resulting in significant differences in microbiota composition between the three sites.

Due to initial difficulties extracting total DNA from the rectal swabs, which required many attempts to refine and validate the process, only 32 of 109 samples yielded DNA of sufficient quality and quantity to assess the microbiota. Despite this, the distribution of the swabs from which total DNA was extracted successfully varied sufficiently to allow statistical analyses to be performed between age groups (pups and yearlings) and also between pups born on three differing natal terrestrial habitats. The optimization process involved two bead types (0.7 mm dry beads and 0.1 mm silica beads) used for bead beating the swabs at the initial stages of extraction using the PowerFecal® DNA isolation kit. The vortexing process before the addition of the C2 solution was also changed to optimize the extraction (max speed for 10 min when using the 0.7 mm beads from the kit and 30 s at 6 m/s in a Fast‐Prep® machine (Thermo) for the 0.1 silica beads extraction). The extraction efficiency was analyzed by Nanodrop quantification and 260:280 and 260:230 ratios. Further, the addition of proteinase K treatment was assessed and determined to improve extraction efficiencies. From these optimization experiments, the finalized method was used as described in Section 2. Future studies will benefit from using the validated technique for total DNA extraction described here to maximize data from samples and also allow direct comparison with this population of gray seals. Further improvements in the preservation of the samples should also be considered in future studies, with samples stored in DNA preservation buffer (to prevent further growth of microbes) rather than frozen in media, as this may have affected the composition and diversity of the microbiota in the samples.

Although 14 different bacterial genera were identified in pup and yearling samples at a limit of >1% abundance, the microbiota of pups was less diverse than that of yearlings, although this was only significant at the 10% level (*p* = 0.62). This difference in diversity is not surprising given the restricted terrestrial habitat of the pups versus the exposure of the yearlings to various prey, each with their bacterial microbiota, and that of the marine environment as a whole. This is exemplified by the higher amount of *Oceaniverga*, a bacterial genus that favors a saline environment, in the microbiota of yearlings versus pups as the former have had much greater exposure to the sea. The failure to reach statistical significance in this difference in microbial diversity in our study may be due to the low number of yearling samples for which sequence data were obtained. Analysis of a larger sample set is required to determine this definitively.

The genus *Fusobacterium* dominated the microbiota of both pups and yearlings sampled on the Isle of May. This is consistent with previous studies investigating the rectal fecal microbiota of seals and sea lions as Fusobacteria was one of the most prevalent phyla, along with Firmicutes, Proteobacteria, Bacteroidetes, and in the study of sea lions, Actinobacteria (Banks et al., 2014; Bik et al., 2016; Delport et al., 2016; Glad et al., 2010; Nelson et al., 2013; Numberger et al., 2016). Both yearling and pup groups also shared microbiota in the genus Bisgaardia, a known zoonotic pathogen that can cause seal finger in humans (Sundeep & Cleeve, 2011), the genera Campylobacter and to some extent *Alistipes*, identified in the lumen of the human colon (Parker et al., 2020).

The presence of the genus *Campylobacter* confirmed similar findings in our previous study using these same samples (Baily et al., 2015). However, in the previous study *Campylobacter* spp. were cultured from 51% of live seal pups but none from yearling seals. This apparent discrepancy was probably due to the vastly different methods used; PCR‐based analysis in the present study to detect all *Campylobacter* spp. versus microbiological culture with media highly selective for specific species of *Campylobacter* known to be pathogenic to humans in the previous study. The isolates identified in our previous study were *Campylobacter jejuni, C. coli*, and *C. lari* (Baily et al., 2015) and are all primarily associated with human disease, with *C. jejuni* being one of the most common causes of gastroenteritis in humans (Acheson & Allos, 2001). The authors concluded that the species and strains of *Campylobacter* isolated were likely indicators of pollution originating from human sewage (Baily et al., 2015). Although interactions between commensal and pathogenic bacteria have the potential to influence the overall microbiota composition (Nelson et al., 2015), preliminary analysis showed no significant correlation detected between pups identified by PCR as having *Campylobacter* and an altered microbiota when compared with those samples where this genus was not detected (data not shown). However, future studies should review this potential association and include identifying the precise *Campylobacter* spp. found.

The microbiota of the seal pups was significantly elevated in the genera *Escherichia/Shigella* and *Clostridium* (sensu stricto) compared with yearlings. As well as being a common commensal, *Escherichia/Shigella* are well‐characterized pathogens of humans, and one of the main causes of bacterial diarrhea (Khalil et al., 2018) but known to be more common in the young of many mammalian species (Chung et al., 2012). This genus is, typically, more prevalent in the microbiota of young mammals or those yet to mature and less abundant in mature mammals with a healthy, stable microbiome (Castaño‐Rodríguez et al., 2018, Tian et al., 2020).

The genus *Clostridium* (sensu stricto), of the family Clostridiaceae, is a major component of the fecal microbiota of human infants and is strongly associated with infancy food allergies, for which it has been proposed as a potential biomarker (Ling et al., 2014), and atopic dermatitis in early childhood (Penders et al., 2013). Although such diseases have not been recognized in seals, the genus *Clostridium* was significantly more abundant in pups than yearlings supports the importance in humans of transition to a mature microbiome with respect to health.

Yearling seal microbiomes were elevated in the genus *Alloprevotella*, a member of the family Prevotellaceae. *Prevotella*, another genus within this family, is known to be a marker for the transition from milk to prey of weaned, independently feeding seals (Stoffel et al., 2020), and therefore this difference found between yearlings and pups is an expected finding. Oceaniverga, Fournierella, and Ruminococcaceae UCG‐005 ASVs were all more abundant in yearlings and this is probably, as mentioned above, a reflection of dietary resources as pups have a restricted diet of milk compared to yearlings that forage widely in the open sea and intertidal areas. This is supported by previous studies that found a significant relationship between diet, gut microbiota composition, and Operational Taxonomic Units (Pacheco‐Sandoval et al., 2019) and that the gut microbiota of fish, mammalian livestock species, and of other animals that forage, manifest greater microbial diversity than those fed from artificial or concentrate sources (Dhanasiri et al., 2011; Ellison et al., 2014; Kohl & Dearing, 2014; Nelson et al., 2013; Sanders et al., 2015). Furthermore, Delport et al. (2016) found that Clostridiaceae and Ruminococcaceae were more abundant in free‐living Australian sea lions (*Neophoca cinerea*) compared with captive ones and these phyla contributed most to the average dissimilarity between groups. Comparison of the diets showed wild animals consumed a wider range of food that included a number of species with chitinous body parts including small crustaceans, rock lobster, and cephalopods, such as cuttlefish, octopus, and squid (Gales & Cheal, 1992) compared to captive animals, which were fed almost entirely fresh or frozen fish (McIntosh et al., 2007).

Our study found no significant effect of sex on microbiota composition, which is consistent with previous studies that found mostly negligible or no effect based on sex in free‐living populations (Bobbie et al., 2017; Maurice et al., 2015; Tung et al., 2015). However, sex confounding effects may be present at more subtle levels, being masked by the possible effects of external factors such as diet or environment on gut microbial communities. In post‐weaned northern elephant seals (*Mirounga angustirostris*), Stoffel et al. (2020) found sex to be a strong and early determinant of gut microbiome composition, but not diversity and therefore is in contrast to our study. It is not clear why we did not find a difference between the sexes as seen in Northern elephant seals. In both species, males are slightly larger at birth and weaning (Fedak & Anderson, 1982; Kretzmann et al., 1993) although the major growth and dimorphic changes occur mainly during the first year of life, once animals have finally departed from their breeding sites. However, differences in the colony environment and habitat, as well as early exploratory behavioral variation between the sexes that differ between the species, could be factors. Therefore, our preliminary study suggests that the diversity of seal microbiomes is age‐dependent (with lower diversity seen in pups relative to yearlings). Although not strictly statistically significant, this pattern of lower diversity in younger animals with developing microbiotas is consistent with those seen in a range of other mammal species studied as animals mature beyond their first year of life (Hopkins et al., 2002, Koenig et al., 2011, Mariat et al., 2009, Stoffel et al., 2020, Yatsunenko et al., 2012).

This study also revealed that gray seal pup rectal microbiomes may be influenced by the terrestrial substrate they are exposed to as neonates. Although not definitive, these differences in the composition of the microbiota in fecal swabs from pups collected from the three different locations (PH, RR, and TS) were highly significant. Given the notably differing substrates in each of these three chosen locations; a tidal rocky boulder beach (PH), nontidal, stagnant rocky pools (RR), and a muddy/grassy slope (TS), seal pups will have been exposed to different environmental microbes which will have been ingested either directly or during suckling from contamination of the teats of their respective dams. In this study, the influence of anthropogenic pressure between the three locations was unlikely to affect results as all the sites were distant from boat mooring sites/human habitation and did not have any meaningful footfall.

Further detailed work is required to determine if these significantly differing microbiomes have any notable effect on long‐term survival given the high mortality rate during the first year of life (Hall et al., 2001). However, there will be multiple confounding factors affecting survival, including intrinsic and extrinsic factors (Grosser et al., 2019) so any study will need to account for all of these.

The presence of the genera *Campylobacter*, *Escherichia/Shigella*, and *Clostridium* sensu stricto could be included in a quality index measuring aquatic mammalian health, and/or the marine environment, in the Firth of Forth and potentially elsewhere. However, yearling and adult seals do have, what appears to be, host‐adapted species of *Campylobacter* (Foster et al., 2020; Gilbert et al., 2017, 2018), but which cause little if any disease and are not of anthropogenic origin. Therefore, *Campylobacter* spp. and probably *Salmonella* also, present in the microbiomes of free‐living wild animals will need to be identified to species and possibly strain level if they are to be indicative of anthropogenic origin and pollution.

Marine mammals are considered to be sentinel species of the ocean as they appear to respond rapidly to ocean disturbances and pathogens similar to humans (Bossart, 2011). Several studies have examined the connections between the community composition of the microbiome and animal health including Apprill et al. (2014), although more detailed studies are still required to fully understand specific correlations.

Future studies should evaluate the potential biomarkers discovered in this pilot study, specifically in pups, and understand how they correlate with age, physiological development, and adaptation of the seals to their environment, particularly in respect of climate change. Due to the immature microbiota and immune system (Fukuda et al., 2011; Maynard et al., 2012; Round & Mazmanian, 2009), there is likely to be less competition between potential pathogens and the commensal microbiota in young pups when compared to yearlings as the latter have a more mature and stable microbiome which can better out‐compete pathogens.

In light of this study, we would expect larger‐scale experiments, with increased statistical power, to identify and validate specific genera as indicators of the health of seal colonies and/or marine health more broadly, and such a tool could add to the long‐term conservation management of marine habitats. However, the species of pinniped and specific populations studied would need to be chosen carefully, taking into account their species‐related life history and foraging patterns, to ensure they are representative of the marine environment being assessed.

## AUTHOR CONTRIBUTIONS

**Craig Watkins**: Conceptualization (equal), data curation (lead), formal analysis‐supporting, funding acquisition‐supporting, investigation (equal), methodology (equal), project administration (equal), resources‐supporting, software‐supporting, supervision (lead), validation‐supporting, visualization‐supporting, writing—original draft (lead), writing—review & editing (lead). **Taylor Gaines**: Conceptualization‐supporting, data curation (equal), formal analysis (equal), funding acquisition‐supporting, methodology‐supporting, resources‐supporting, software‐supporting, writing—review & editing‐supporting. **Fiona Strathdee**: Data curation‐supporting, methodology‐supporting, supervision‐supporting, writing—review & editing‐supporting. **Johanna Bailey**: Conceptualization‐supporting, data curation‐supporting, formal analysis‐supporting, investigation‐supporting, methodology‐supporting, resources‐supporting, writing—review & editing‐supporting. **Eleanor Watson**: Data curation‐supporting, investigation‐supporting, resources‐supporting, validation‐supporting, writing—review & editing‐supporting. **Ailsa Hall**: Investigation‐supporting, resources‐supporting, writing—review & editing‐supporting. **Andrew Free**: Conceptualization‐supporting, data curation‐supporting, formal analysis (lead), funding acquisition (lead), investigation (equal), methodology (equal), project administration (equal), resources (equal), software (lead), supervision (equal), validation (equal), visualization (equal), writing—original draft‐supporting, writing—review & editing (equal). **Mark Dagleish**: Conceptualization‐supporting, data curation‐supporting, investigation‐supporting, methodology‐supporting, resources (equal), writing—original draft‐supporting, writing—review & editing (equal).

## CONFLICTS OF INTEREST

None declared.

## ETHICS STATEMENT

All sampling of live animals was authorized by the University of St. Andrews Animal Welfare and Ethics Committee and carried out under UK Home Office Project (No. 60/4009) and associated Personal Licenses issued to the Sea Mammal Research Unit (SMRU) under the UK Animals (Scientific Procedures) Act, 1986.

## Data Availability

Sequencing data and metadata are publically available in the European Nucleotide Archive (ENA) at the European Bioinformatics Institute (EBI), under the following study accession numbers: for seal samples PRJEB35901: https://www.ebi.ac.uk/ena/browser/view/PRJEB35901 and for technical control samples PRJEB35983: https://www.ebi.ac.uk/ena/browser/view/PRJEB35983.

## References

[mbo31281-bib-0001] Acheson, D. , & Allos, B. (2001). *Campylobacter jejuni* Infections: Update on emerging issues and trends. Clinical Infectious Diseases, 32(8), 1201–1206. 10.1086/319760 11283810

[mbo31281-bib-0002] Anderson, M. J. , Gorely, R. N. , & Clarke, K. R. (2008). PERMANOVA+ PRIMER: Guide to software and statistical methods. Plymouth, UK, Primer‐E.

[mbo31281-bib-0003] Apprill, A. (2017). Marine animal microbiomes: Toward understanding host–microbiome interactions in a changing ocean. Frontiers in Marine Science, 4, 222. 10.3389/fmars.2017.00222

[mbo31281-bib-0004] Apprill, A. , Robbins, J. , Eren, A. M. , Pack, A. A. , Reveillaud, J. , Matilla, D. , Moore, M. , Niemeyer, M. , Moore, K. T. M. , & Mincer, T. J. (2014). Humpback whale populations share a core skin bacterial community: Towards a health index for marine mammals? PLoS One, 9, e90785. 10.1371/journal.pone.0090785 24671052 PMC3966734

[mbo31281-bib-0005] Baily, J. (2014). *Halichoerus grypus*: The pathology and occurrence of pathogens in Scottish grey seals (Thesis). https://research-repository.st-andrews.ac.uk/handle/10023/4856

[mbo31281-bib-0006] Baily, J. , Foster, G. , Brown, D. , Davison, N. , Coia, J. , Watson, E. , Pizzi, R. , Willoughby, K. , Hall, A. J. , & Dagleish, M. P. (2016). *Salmonella* infection in grey seals (*Halichoerus grypus*), a marine mammal sentinel species: Pathogenicity and molecular typing of *Salmonella* strains compared with human and livestock isolates. Environmental Microbiology, 18(3), 1078–1087. 10.1111/1462-2920.13219 26768299

[mbo31281-bib-0007] Baily, J. , Méric, G. , Bayliss, S. , Foster, G. , Moss, S. , Watson, E. , Pascoe, B. , Mikhail, J. , Pizzi, R. , Goldstone, R. J. , Smith, D. G. , Willoughby, K. , Hall, A. J. , Sheppard, S. K. , & Dagleish, M. P. (2015). Evidence of land‐sea transfer of the zoonotic pathogen *Campylobacter* to a wildlife marine sentinel species. Molecular Ecology, 24(1), 208–221. 10.1111/mec.13001 25401947

[mbo31281-bib-0008] Banks, J. , Cary, S. , & Hogg, I. (2014). Isolated fecal bacterial communities found for Weddell seals, *Leptonychotes weddellii*, at White Island, McMurdo Sound, Antarctica. Polar Biology, 37(12), 1857–1864. 10.1007/s00300-014-1567-x

[mbo31281-bib-0009] Bik, E. , Costello, E. , Switzer, A. , Callahan, B. , Holmes, S. , Wells, R. , Carlin, K. P. , Jensen, E. D. , Venn‐Watson, S. , & Relman, D. A. (2016). Marine mammals harbor unique microbiotas shaped by and yet distinct from the sea. Nature Communications, 7, 10516. 10.1038/ncomms10516 PMC4742810 26839246

[mbo31281-bib-0010] Bobbie, C. B. , Mykytczuk, N. C. S. , & Schulte‐Hostedde, A. I. (2017). Temporal variation of the microbiome is dependent on body region in a wild mammal (*Tamiasciurus hudsonicus*). FEMS Microbiology Ecology, 93(7), fix08. 10.1093/femsec/fix081 28645188

[mbo31281-bib-0011] Bokulich, N. A. , Kaehler, B. D. , Rideout, J. R. , Dillon, M. , Bolyen, E. , Knight, R. , Huttley, G. A. , & Caporaso, J. G. (2018). Optimizing taxonomic classification of marker‐gene amplicon sequences with QIIME 2's q2‐feature‐classifier plugin. Microbiome, 6(1), 90. 29773078 10.1186/s40168-018-0470-z PMC5956843

[mbo31281-bib-0012] Bolyen, E. , Rideout, J. R. , Dillon, M. R. , Bokulich, N. A. , Abnet, C. C. , Al‐Ghalith, G. A. , Alexander, H. , Alm, E. J. , Arumugam, M. , Asnicar, F. , Bai, Y. , Bisanz, J. E. , Bittinger, K. , Brejnrod, A. , Brislawn, C. J. , Brown, C. T. , Callahan, B. J. , Caraballo‐Rodríguez, A. M. , Chase, J. , … Caporaso, J. G. (2019). Reproducible, interactive, scalable and extensible microbiome data science using QIIME 2. Nature Biotechnology, 37(8), 852–857. 10.1038/s41587-019-0209-9 PMC7015180 31341288

[mbo31281-bib-0013] Bonde, R. K. , Aguirre, A. A. , & Powell, J. (2004). Manatees as sentinels of marine ecosystem health: Are they the 2000‐pound canaries? EcoHealth, 1, 255–262. 10.1007/s10393-004-0095-5.2

[mbo31281-bib-0014] Bossart, G. D. (2011). Marine mammals as sentinel species for oceans and human health. Veterinary Pathology, 48(3), 676–690. 10.1177/0300985810388525 21160025

[mbo31281-bib-0015] Budding, A. , Grasman, M. , Eck, A. , Bogaards, J. , Vandenbroucke‐Grauls, C. , van Bodegraven, A. , & Savelkoul, P. (2014). Rectal swabs for analysis of the intestinal microbiota. PLoS One, 9(7), e101344. 10.1371/journal.pone.0101344 25020051 PMC4096398

[mbo31281-bib-0016] Callahan, B. J. , McMurdie, P. J. , Rosen, M. J. , Han, A. W. , Johnson, A. J. , & Holmes, S. P. (2016). DADA2: High‐resolution sample inference from Illumina amplicon data. Nature Methods, 13(7), 581–583. 27214047 10.1038/nmeth.3869 PMC4927377

[mbo31281-bib-0017] Caporaso, J. G. , Lauber, C. L. , Walters, W. A. , Berg‐Lyons, D. , Huntley, J. , Fierer, N. , Owens, S. M. , Betley, J. , Fraser, L. , Bauer, M. , Gormley, N. , Gilbert, J. A. , Smith, G. , & Knight, R. (2012). Ultra‐high‐throughput microbial community analysis on the Illumina HiSeq and MiSeq platforms. The ISME Journal: Multidisciplinary Journal of Microbial Ecology, 6(8), 1621–1624. 10.1038/ismej.2012.8 PMC3400413 22402401

[mbo31281-bib-0018] Castaño‐Rodríguez, N. , Underwood, A. P. , Merif, J. , Riordan, S. M. , Rawlinson, W. D. , Mitchell, H. M. , & Kaakoush, N. O. (2018). Gut microbiome analysis identifies potential etiological factors in acute gastroenteritis. Infection and Immunity, 86(7), 1–13. 10.1128/IAI.00060-18 PMC6013661 29685983

[mbo31281-bib-0019] Chung, H. , Pamp, S. J. , Hill, J. A. , Surana, N. K. , Edelman, S. M. , Troy, E. B. , Reading, N. C. , Villablanca, E. J. , Wang, S. , Mora, J. R. , Umesaki, Y. , Mathis, D. , Benoist, C. , Relman, D. A. , & Kasper, D. L. (2012). Gut immune maturation depends on colonization with a host‐specific microbiota. Cell, 149(7), 1578–1593. 10.1016/j.cell.2012.04.037 22726443 PMC3442780

[mbo31281-bib-0020] Clark, K. , & Warwick, R. (2001). Change in marine communities: An approach to statistical analysis and interpretation (2nd ed.). PRIMER‐E Ltd.

[mbo31281-bib-0022] Cook, P. F. , Reichmuth, C. , Rouse, A. A. , Libby, L. A. , Dennison, S. E. , Carmichael, O. T. , Kruse‐Elliott, K. T. , Bloom, J. , Singh, B. , Fravel, V. A. , & Barbosa, L. (2015). Algal toxin impairs sea lion memory and hippocampal connectivity, with implications for strandings. Science, 350, 1545–1547. 10.1126/science.aac5675.3 26668068

[mbo31281-bib-0023] Delport, T. , Power, M. , Harcourt, R. , Webster, K. , & Tetu, S. (2016). Colony location and captivity influence the gut microbial community composition of the Australian Sea Lion (*Neophoca cinerea*). Applied and Environmental Microbiology, 82(12), 3440–3449. 10.1128/aem.00192-16 27037116 PMC4959163

[mbo31281-bib-0024] Dhanasiri, A. K. , Brunvold, L. , Brinchmann, M. F. , Korsnes, K. , Bergh, O. , & Kiron, V. (2011). Changes in the intestinal microbiota of wild Atlantic cod *Gadus morhua* L. upon captive rearing. Microbial Ecology, 61, 20–30. 10.1007/s00248-010-9673-y 20424834

[mbo31281-bib-0025] Ellison, M. J. , Conant, G. C. , Cockrum, R. R. , Austin, K. J. , Truong, H. , Becchi, M. , Lamberson, W. R. , & Cammack, K. M. (2014). Diet alters both the structure and taxonomy of the ovine gut microbial ecosystem. DNA Research, 21, 115–125. 10.1093/dnares/dst044 24170804 PMC3989484

[mbo31281-bib-0026] Fedak, M. A. , & Anderson, S. S. (1982). The energetics of lactation: Accurate measurements from a large wild mammal, the grey seal (*Halichoerus grypus*). Journal of Zoology, London, 198, 473–479.

[mbo31281-bib-0027] Foster, G. , Baily, J. , Howie, F. , Brownlow, A. , Wagenaar, J. A. , Gilbert, M. J. , Miller, W. G. , Patterson, I. A. , Reid, R. J. , & Dagleish, M. P. (2020). *Campylobacter pinnipediorum* subsp. caledoniensis recovered from abscesses in seals. DAO. 142, 41–46. 10.3354/dao03544 33210610

[mbo31281-bib-0028] Fukuda, S. , Toh, H. , Hase, K. , Oshima, K. , Nakanishi, Y. , Yoshimura, K. , Tobe, T. , Clarke, J. M. , Topping, D. L. , Suzuki, T. , Taylor, T. D. , Itoh, K. , Kikuchi, J. , Morita, H. , Hattori, M. , & Ohno, H. (2011). Bifidobacteria can protect from enteropathogenic infection through production of acetate. Nature, 469(7331), 543–547. 21270894 10.1038/nature09646

[mbo31281-bib-0029] Gales, N. J. , & Cheal, A. J. (1992). Estimating diet composition of the Australian sea‐lion (*Neophoca cinerea*) from scat analysis: An unreliable technique. Wildlife Research, 19, 447–456. 10.1071/WR9920447

[mbo31281-bib-0030] Gilbert, M. J. , Miller, W. G. , Leger, J. S. , Chapman, M. H. , Timmerman, A. J. , Duim, B. , Foster, G. , & Wagenaar, J. A. (2017). *Campylobacter pinnipediorum* sp. nov., isolated from pinnipeds, comprising *Campylobacter pinnipediorum* subsp*. pinnipediorum* subsp. nov. and *Campylobacter pinnipediorum* subsp. *Caledonicus* subsp. International Journal of Systematic and Evolutionary Microbiology, 67(6), 1961–1968. 10.1099/ijsem.0.001894 28629508

[mbo31281-bib-0031] Gilbert, M. J. , Zomer, A. L. , Timmerman, A. J. , Spaninks, M. P. , Rubio‐García, A. , Rossen, J. W. , Duim, B. , & Wagenaar, J. A. (2018). *Campylobacter blaseri* sp. nov., isolated from common seals (*Phoca vitulina*). International Journal of Systematic and Evolutionary Microbiology, 68(5), 1787–1794. 10.1099/ijsem.0.002742 29624164

[mbo31281-bib-0032] Glad, T. , Kristiansen, V. F. , Nielsen, K. M. , Brusetti, L. , Wright, A. G. , & Sundset, M. A. (2010). Ecological characterization of the colonic microbiota in arctic and sub‐arctic seals. Microbial Ecology, 60, 323–330. 10.1007/s00248-010-9690-x 20523986

[mbo31281-bib-0033] Godfray, H. C. J. , Stephens, A. E. A. , Jepson, P. D. , Jobling, S. , Johnson, A. C. , Matthiessen, P. , Sumpter, J. P. , Tyler, C. R. , & McLean, A. R. (2019). A restatement of the natural science evidence base on the effects of endocrine disrupting chemicals on wildlife. Proceedings. Biological sciences/The Royal Society, 286(1897), 20182416. 10.1098/rspb.2018.2416 PMC6408895 30963852

[mbo31281-bib-0034] Grosser, S. , Sauer, J. , Paijmans, A. J. , Caspers, B. A. , Forcada, J. , Wolf, J. B. W. , & Hoffman, J. I. (2019). Fur seal microbiota are shaped by the social and physical environment, show mother–offspring similarities and are associated with host genetic quality. Molecular Ecology, 28, 2406–2422. 10.1111/mec.15070 30849214

[mbo31281-bib-0035] Gulland, F. M. (1999). Stranded seals: Important sentinels. Journal of the American Veterinary Medical Association, 214(8), 1191–1192. 10212681

[mbo31281-bib-0036] Hall, A. J. , McConnell, B. J. , & Barker, R. J. (2001). Factors affecting first‐year survival in grey seals and their implications for life history strategy. Journal of Animal Ecology, 70(1), 138–149. 10.1111/j.1365-2656.2001.00468.x

[mbo31281-bib-0037] Hanning, I. , & Diaz‐Sanchez, S. (2015). The functionality of the gastrointestinal microbiome in non‐human animals. Microbiome, 3(1), 51. 26552373 10.1186/s40168-015-0113-6 PMC4640220

[mbo31281-bib-0038] Hopkins, M. J. , Sharp, R. , & Macfarlane, G. T. (2002). Variation in human intestinal microbiota with age. Digestive and Liver Disease, 34(Suppl 2), S12–S18. 10.1016/S1590-8658(02)80157-8 12408433

[mbo31281-bib-0039] Jepson, P. D. , Bennett, P. M. , Deaville, R. , Allchin, C. R. , Baker, J. R. , & Law, R. J. (2005). Relationships between polychlorinated biphenyls and health status in harbor porpoises (*Phocoena phocoena*) stranded in the United Kingdom. Environmental Toxicology and Chemistry, 24(1), 238–248. 10.1897/03-663.1 15683190

[mbo31281-bib-0040] Jessup, D. , Miller, M. , Ames, J. , Harris, M. , Kreuder, C. , Conrad, P. A. , & Mazet, J. (2004). Southern sea otter as a sentinel of marine ecosystem health. Eco‐Health, 1, 239–245. 10.1007/s10393-004-0093-7.4

[mbo31281-bib-0041] Khalil, I. A. , Troeger, C. , Blacker, B. F. , Rao, P. C. , Brown, A. , Atherly, D. E. , Brewer, T. G. , Engmann, C. M. , Houpt, E. R. , Kang, G. , Kotloff, K. L. , Levine, M. M. , Luby, S. P. , MacLennan, C. A. , Pan, W. K. , Pavlinac, P. B. , Platts‐Mills, J. A. , Qadri, F. , Riddle, M. S. , … Reiner, R. C., Jr. (2018). Morbidity and mortality due to shigella and enterotoxigenic *Escherichia coli* diarrhoea: The Global Burden of Disease Study 1990–2016. The Lancet, 18(11), 1229–1240. 10.1016/S1473-3099(18)30475 30266330 PMC6202441

[mbo31281-bib-0042] Koenig, J. E. , Spor, A. , Scalfone, N. , Fricker, A. D. , Stombaugh, J. , Knight, R. , Angenent, L. T. , & Ley, R. E. (2011). Succession of microbial consortia in the developing infant gut microbiome. Proceedings of the National Academy of Sciences of the United States of America, 108(Suppl 1), 4578–4585. 10.1073/pnas.1000081107 20668239 PMC3063592

[mbo31281-bib-0043] Kohl, K. D. , & Dearing, M. D. (2014). Wild‐caught rodents retain a majority of their natural gut microbiota upon entrance into captivity. Environmental Microbiology Reports, 6, 191–195. 10.1111/1758-2229.12118 24596293

[mbo31281-bib-0044] Kretzmann, M. B. , Costa, D. P. , & Le Boeuf, B. J. (1993). Maternal energy investment in elephant seal pups: Evidence for sexual equality? The American Naturalist, 141(3), 466–480. 10.1086/285484 19426016

[mbo31281-bib-0045] Ling, Z. , Li, Z. , Xia Liu, X. , Yiwen Cheng, Y. , Yueqiu Luo, Y. , Xiaojuan Tong, X. , Li Yuan, L. , Yuezhu Wang, Y. , Jinbo Sun, J. , Lanjuan Li, L. , & Charlie Xiang, C. (2014). Altered fecal microbiota composition associated with food allergy in infants. Applied and Environmental Microbiology, 80(8), 2546–2554. 24532064 10.1128/AEM.00003-14 PMC3993190

[mbo31281-bib-0046] Mandal, S. , Van Treuren, W. , White, R. A. , Eggesbø, M. , Knight, R. , & Peddada, S. D. (2015). Analysis of composition of microbiomes: A novel method for studying microbial composition. Microbial Ecology in Health and Disease, 26(1), 27663. 26028277 10.3402/mehd.v26.27663 PMC4450248

[mbo31281-bib-0047] Mariat, D. , Firmesse, O. , Levenez, F. , Guimarăes, V. , Sokol, H. , Doré, J. , Corthier, G. , & Furet, J. ‐P. (2009). The firmicutes/bacteroidetes ratio of the human microbiota changes with age. BMC Microbiology, 9, 123. 10.1186/1471-2180-9-123 19508720 PMC2702274

[mbo31281-bib-0048] Maurice, C. F. , Knowles, S. C. L. , Ladau, J. , Pollard, K. S. , Fenton, A. , Pedersen, A. B. , & Turnbaugh, P. J. (2015). Marked seasonal variation in the wild mouse gut microbiota. ISME Journal, 9, 2423–2434. 10.1038/ismej.2015.53 26023870 PMC4611506

[mbo31281-bib-0049] Maynard, C. L. , Elson, C. O. , Hatton, R. D. , & Weaver, C. T. (2012). Reciprocal interactions of the intestinal microbiota and immune system. Nature, 489(7415), 231–241. 22972296 10.1038/nature11551 PMC4492337

[mbo31281-bib-0050] McIntosh, R. R. , Page, B. , & Goldsworthy, S. D. (2007). Dietary analysis of regurgitates and stomach samples from free‐living Australian sea lions. Wildlife Research, 33, 661–669. 10.1071/wr06025

[mbo31281-bib-0051] Moore, S. (2008). Marine mammals as ecosystem sentinels. Journal of Mammalogy, 89(3), 534–540. 10.1644/07-MAMM-S-312R1.1.5

[mbo31281-bib-0052] Nelson, T. , Rogers, T. , & Brown, M. (2013). The gut bacterial community of mammals from marine and terrestrial habitats. PLoS One, 8(12), e83655. 10.1371/journal.pone.0083655 24386245 PMC3875473

[mbo31281-bib-0053] Nelson, T. M. , Apprill, A. , Mann, J. , Rogers, T. L. , & Brown, M. V. (2015). The marine mammal microbiome: Current knowledge and future directions. Microbiology Australia, 36, 8–13. 10.1071/MA15004

[mbo31281-bib-0054] Numberger, D. , Herlemann, D. P. R. , Jürgens, K. , Dehnhardt, G. , & Schulz‐Vogt, H. (2016). Comparative analysis of the fecal bacterial community of five harbor seals (*Phoca vitulina*). Microbiology Open 2016, 5(5), 782–792. 10.1002/mbo3.369 PMC5061715 27734626

[mbo31281-bib-0055] Pacheco‐Sandoval, A. , Schramm, Y. , Heckel, G. , Brassea‐Pe'rez, E. , Martinez‐Porchas, M. , & Lago‐Lesto'n, A. (2019). The Pacific harbor seal gut microbiotain Mexico: Its relationship with diet and functional inferences. PLoS One, 14(8), e0221770. 10.1371/journal.pone.0221770 31465508 PMC6715212

[mbo31281-bib-0056] Parker, B. J. , Wearsch, P. A. , Veloo, A. C. M. , & Rodriguez‐Palacios, A. (2020). The genus *Alistipes*: Gut bacteria with emerging implications to inflammation, cancer, and mental health. Frontiers in immunology, 11, 906. 10.3389/fimmu.2020.00906 32582143 PMC7296073

[mbo31281-bib-0057] Penders, J. , Gerhold, K. , Stobberingh, E. E. , Thijs, C. , Zimmermann, K. , Lau, S. , & Hamelmann, E. (2013). Establishment of the intestinal microbiota and its role for atopic dermatitis in early childhood. Journal of Allergy and Clinical Immunology, 132, 601–607. 23900058 10.1016/j.jaci.2013.05.043

[mbo31281-bib-0058] Reddy, M. L. , Dierauf, L. A. , & Gulland, F. M. D. (2001). Marine mammals as sentinels of ocean health. In L. A. Dierauf , & F. M. D. Gulland (Eds.), CRC handbook of marine mammal medicine (pp. 3–13). CRC Press.

[mbo31281-bib-0059] Reeves, R. R. , Stewart, B. S. , Clapham, P. J. , Powell, J. A. , & Folkens, P. A. (2002). Sea mammals of the world (pp. 138–141). A & C Black Publishers Ltd.

[mbo31281-bib-0060] Round, J. L. , & Mazmanian, S. K. (2009). The gut microbiota shapes intestinal immune responses during health and disease. Nature Reviews Immunology, 9(5), 313–323. 10.1038/nri2515 PMC4095778 19343057

[mbo31281-bib-0062] Sanders, J. , Beichman, A. , Roman, J. , Scott, J. J. , Emerson, D. , McCarthy, J. J. , & Girguis, P. R. (2015). Baleen whales host a unique gut microbiome with similarities to both carnivores and herbivores. Nature Communications, 6, 8285. 10.1038/ncomms9285 PMC4595633 26393325

[mbo31281-bib-0063] Smith, S. , Chalker, A. , Dewar, M. , & Arnould, J. (2013). Age‐related differences revealed in Australian fur seal *Arctocephalus pusillus doriferus* gut microbiota. FEMS Microbiology Ecology, 86(2), 246–255. 10.1111/1574-6941.12157 23746080

[mbo31281-bib-0064] Smout, S. , King, R. , & Pomeroy, P. (2011). Integrating heterogeneity of detection and mark loss to estimate survival and transience in UK grey seal colonies. Journal of Applied Ecology, 48(2), 364–372.

[mbo31281-bib-0065] Stoffel, M. A. , Acevedo‐Whitehouse, K. , Morales‐Durán, N. , Grosser, S. , Chakarov, N. , Krüger, O. , Nichols, H. J. , Elorriaga‐Verplancken, F. R. , & Hoffman, J. I. (2020). Early sexual dimorphism in the developing gut microbiome of northern elephant seals. Molecular Ecology, 29, 2109–2122. 10.1111/mec.15385 32060961

[mbo31281-bib-0066] Sundeep, S. , & Cleeve, V. (2011). Isolation of *Bisgaardia hudsonensis* from a seal bite. Case report and review of the literature on seal finger. The Journal of Infection, 63(1), 86–88. 10.1016/j.jinf.2011.04.006 21565407

[mbo31281-bib-0067] Tian, J. , Du, J. , Han, J. , Song, X. , & Lu, Z. (2020). Age‐related differences in gut microbial community composition of captive spotted seals (*Phoca largha*). Marine Mammal Science, 36, 1231–1240. 10.1111/mms.12728

[mbo31281-bib-0068] Tung, J. , Barreiro, L. B. , Burns, M. B. , Grenier, J.‐C. , Lynch, J. , Grieneisen, L. E. , Altmann, J. , Alberts, S. C. , Blekhman, R. , & Archie, E. A. (2015). Social networks predict gut microbiome composition in wild baboons. eLife, 4, e05224. 10.7554/eLife.05224 PMC4379495 25774601

[mbo31281-bib-0069] Watkins, C. A. , Bartley, D. J. , Ergün, B. G. , Yıldızhan, B. , Ross‐Watt, T. , Morrison, A. A. , Rosales Sanmartín, M. J. , Strathdee, F. , Andrews, L. , & Free, A. (2021). Interactions between *Teladorsagia circumcincta* infections and microbial composition of sheep with or without successful monepantel treatment: A preliminary study. Ruminants, 1, 31–45. 10.3390/ruminants1010003

[mbo31281-bib-0070] Wells, R. S. , Rhinehart, H. , Hansen, L. , Sweeney, J. , Townsend, F. , Stone, R. , Casper, D. R. , Scott, M. , Hohn, A. , & Rowles, T. (2004). Bottlenose dolphins as marine ecosystems sentinels: Developing a health monitoring system. Eco‐Health, 1, 246–254. 10.1007/s10393-004-0094-6

[mbo31281-bib-0071] Yatsunenko, T. , Rey, F. E. , Manary, M. J. , Trehan, I. , Dominguez‐Bello, M. G. , Contreras, M. , Magris, M. , Hidalgo, G. , Baldassano, R. N. , Anokhin, A. P. , Heath, A. C. , Warner, B. , Reeder, J. , Kuczynski, J. , Caporaso, J. G. , Lozupone, C. A. , Lauber, C. , Clemente, J. C. , Knights, D. , … Ordon, J. I. (2012). Human gut microbiome viewed across age and geography. Nature, 486, 222–227. 10.1038/nature11053 22699611 PMC3376388

